# A case of epithelioid cell granulomas arising at the margin of lung resection, with high accumulation on 18F‐fluorodeoxyglucose‐positron emission tomography

**DOI:** 10.1002/rcr2.830

**Published:** 2021-08-17

**Authors:** Shinnosuke Yokotsuka, Shugo Uematsu, Momoka Okada, Shinnosuke Takamiya, Shinichi Ohashi, Yoko Tanaka, Kosuke Suzuki, Akihiko Kitami, Tetsuo Nemoto

**Affiliations:** ^1^ Department of Medicine Showa University School of Medicine Tokyo Japan; ^2^ Respiratory Disease Center Showa University Northern Yokohama Hospital Yokohama Japan; ^3^ Pathology and Laboratory Medicine Showa University Northern Yokohama Hospital Yokohama Japan

**Keywords:** granuloma, high FDG‐PET accumulation, margin of lung resection, *Mycobacterium tuberculosis*, recurrence

## Abstract

It is important to distinguish tumour recurrence from other conditions that could show high accumulation on 18F‐fluorodeoxyglucose‐positron emission tomography (FDG‐PET). We describe the case of a 78‐year‐old woman who underwent partial resection of the left lower lung lobe for carcinoid treatment 20 years previously. Five years earlier, chest radiography revealed an abnormal shadow, and chest computed tomography (CT) showed partial atelectasis in the left S8. Periodical CT showed that the atelectasis had developed into a mass. The patient was referred to our hospital. A mass of 45 mm diameter was detected on CT and it had a maximum standardized uptake value of 8.91 on FDG‐PET. We suspected recurrence and performed surgery. Pathological examination revealed epithelioid cell granuloma (maximum diameter, 25 mm) with necrosis. Tissue culture showed no evidence of *Mycobacterium tuberculosis*. However, serum anti‐MAC antibody level was elevated, suggesting epithelioid cell granuloma caused by non‐tuberculous Mycobacterium infection.

## Graphical abstract

It seems crucial to differentiate tumour recurrence from other conditions that could show high accumulation on 18F‐fluorodeoxyglucose‐positron emission tomography. We present a case of epithelioid cell granulomas arising at the margin of lung resection for carcinoid treatment 20 years previously.

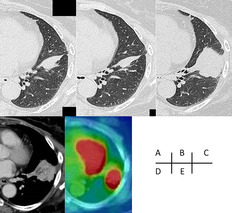

## INTRODUCTION

Malignant tumour recurrence should be suspected when a mass arising at the dissection margin of lung resection for a malignant tumour shows high accumulation on 18F‐fluorodeoxyglucose‐positron emission tomography (FDG‐PET). However, it is critical to acknowledge that several other diseases, besides malignancy, present with high accumulation on FDG‐PET.^1^ We experienced a case where epithelioid cell granulomas that were identified as areas with hyper‐accumulation on FDG‐PET were detected near the resection margin of pulmonary carcinoids.

## CASE REPORT

**FIGURE 1:**
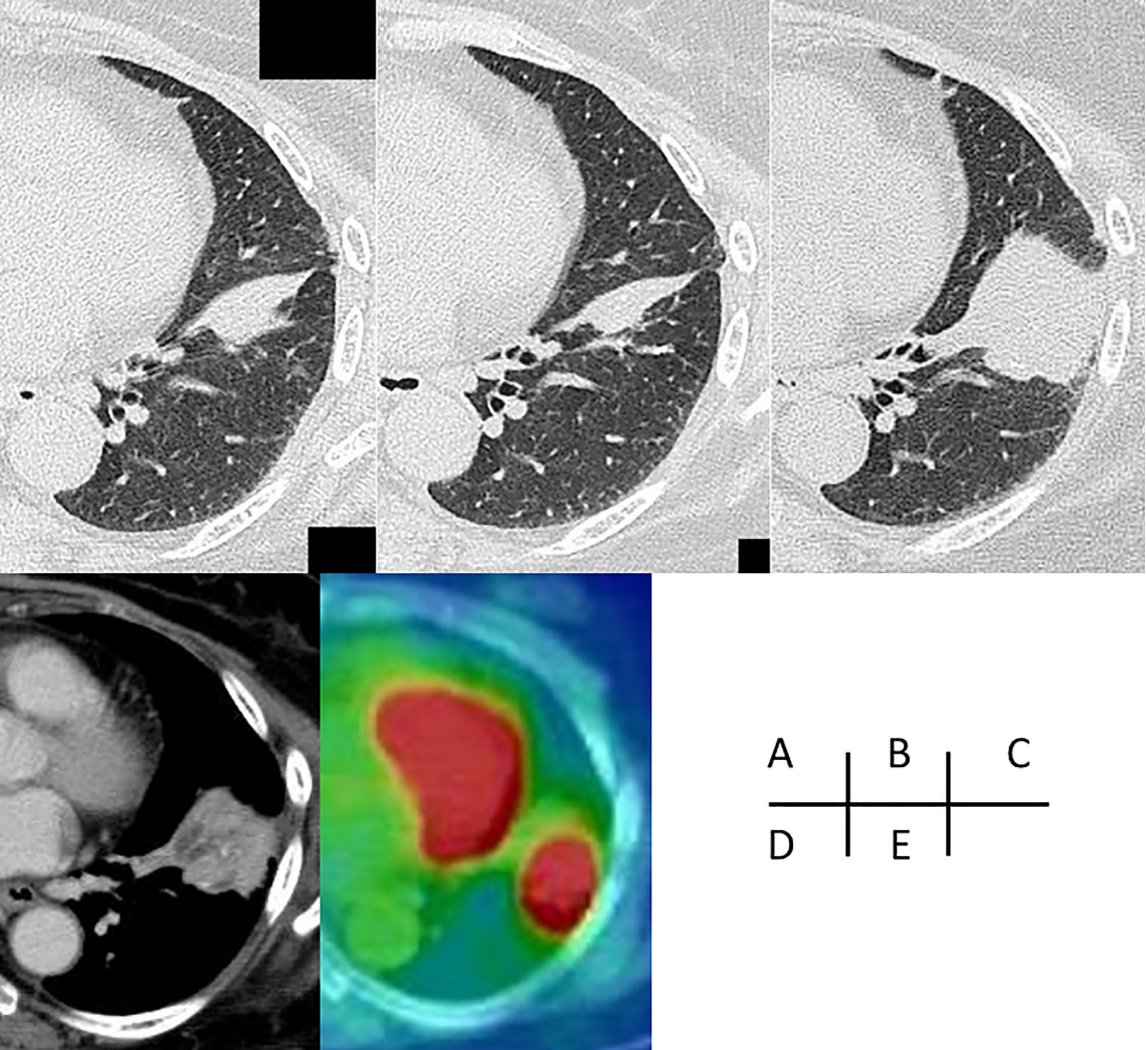
Temporal changes to the mass on chest computed tomography (CT) (A, 3 years previously; B, 1 year previously; C, preoperative image). Preoperative CT shows a mass, 45 × 37 × 32 mm, and contrast effects are seen on the margins of the mass (C, D). The mass shows a maximum standardized uptake value of 8.91 on 18F‐fluorodeoxyglucose‐positron emission tomography (E)

**FIGURE 2:**
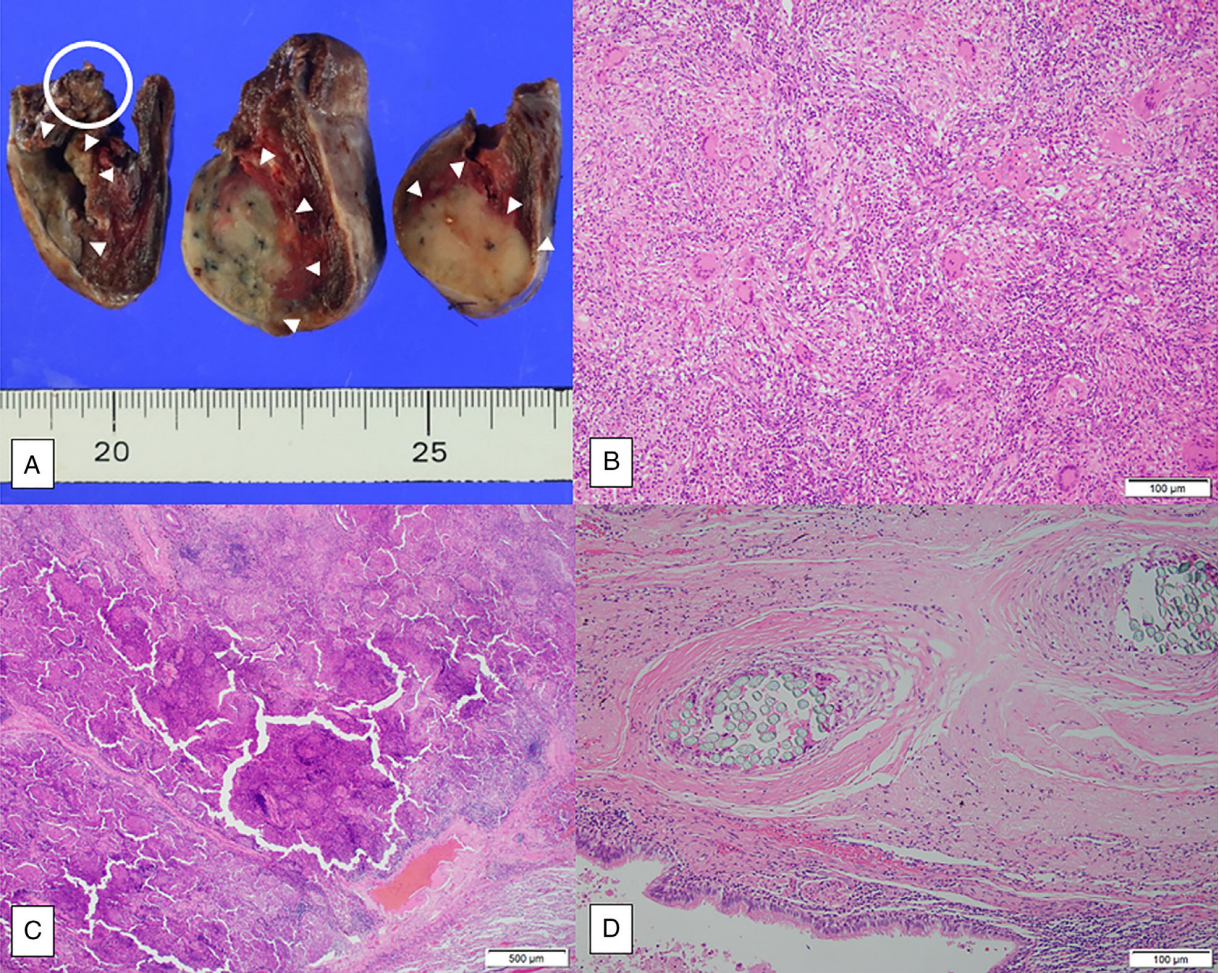
Gross findings indicate a 25 × 18 mm mass (arrowhead) and sutures (white circle). These two lesions are discontinuous (A). Histological examination shows that the mass was formed by epithelioid cell granulomas, which are composed of multinucleated giant cells, and foamy histiocytic infiltration (B), and necrosis can also be detected (C). The suture formed a 10‐mm suture granuloma (D)

We describe a 78‐year‐old woman who underwent partial resection of the left lower lobe due to lung carcinoid 20 years previously. Details of the surgery were unavailable. Five years before the time of this report, when she underwent an annual medical check‐up, an abnormal shadow was noted in the left lower lung on chest radiography. A chest computed tomography (CT) scan revealed partial atelectasis in the left S8. Two years later, CT showed similar findings (Figure 1A). CT was repeated 2 years subsequently and it showed no obvious changes (Figure 1B). However, CT performed 1 month before the patient presented to our hospital revealed that the atelectasis had developed into a mass, suggesting malignancy. Thus, she was referred to our hospital for further investigation and treatment. In our institution, a CT scan showed a 45 × 37 × 32 mm mass with fine calcification in the left S8 (Figure 1C). Contrast‐enhanced CT showed enhancement around the mass (Figure 1D). There were no suspicious findings of malignancy or infection in other lung fields or other organs. The patient had no subjective symptoms or previous episodes of pneumonia or other infections. White blood cell count and C‐reactive protein level were within normal limits, and the levels of tumour markers for lung cancer (carcinoembryonic antigen, cytokeratin 19 fragment and pro‐gastrin‐releasing peptide) were also within normal limits. Bronchoscopy was unable to visualize the mass due to B8 obstruction. FDG‐PET/CT showed accumulation consistent with the mass, with a maximum standardized uptake value (SUVmax) of 8.91 (Figure 1E). Based on these results, we performed surgery to confirm pulmonary malignant tumour, including the recurrence of carcinoid, although it had been a long time since carcinoid resection was performed. After intraoperative diagnosis through fine‐needle biopsy, the mass was suspected to be epithelioid cell granuloma; thus, the S8 segment was resected. Pathological examination revealed a large mass of 25 mm diameter and a 10‐mm suture granuloma centred on the suture (Figure 2A). Histologically, the mass was an epithelioid cell granuloma with necrosis (Figure 2B,C). No fungi were detected by periodic acid‐Schiff or Grocott's methenamine‐silver staining. Although no organism was identified by acid‐fast staining of tissue sections and no acid‐fast bacillus was cultured, it was suspected that the granuloma formation was due to an atypical acid‐fast bacillus infection because of a high serum anti‐MAC antibody level. The suture granuloma, with a braided non‐absorbent suture at its core, was formed by minimal inflammatory cell infiltration, without an abscess (Figure 1, 2). The epithelioid cell and suture granulomas were discontinuous.

## DISCUSSION

Besides malignant tumours, some clinical conditions can show high FDG‐PET accumulation. These include sarcoidosis, rheumatoid nodules, chronic granulomatous disease, tuberculosis and opportunistic infections.^1^ The average SUVmax of non‐tuberculous mycobacteriosis suspected to be caused by a primary pulmonary malignant tumour was 4.9.^2^ However, some cases of higher accumulation have been reported, and it is difficult to differentiate malignant tumours from benign lesions using FDG‐PET/CT.^1^

When a mass appears at the margin of a lung tumour resection, it is especially crucial to distinguish tumour recurrence from changes associated with the surgery. In recent years, granuloma formation at the stump of lung resection that was performed via stapling has been reported.^3^, ^4^ Differences in the shape of shadows arising from the staple line^3^ and evaluation using diffusion‐weighted magnetic resonance imaging (DWI)^4^ have been considered helpful for differentiating tumour recurrence from surgery‐related granulomas. DWI may be useful for differentiating masses arising from margins of resection when stapling was not performed, as in this case.

There are only a few reports on the occurrence of suture granuloma after lung resection. It is assumed that the surgeon carefully selects the suturing material and the lung tissue is not prone to excessive foreign body reaction to the suture material. However, in a previous case, abscess formation was clinically significant, suggesting suture infection, excessive foreign body reaction and infection through the bronchi connected to the surgical site.^5^ Conventionally, it is assumed that stapling would cause less foreign body reaction than suturing. However, it was suggested that foreign body reaction to staple material, anatomical tissue changes caused by stapling and impairment of ventilation and blood flow might cause granuloma formation.^3^ In this case, the suture granuloma was discontinuous with the epithelioid cell granuloma; thus, we assumed that the anatomical changes and the impairment of ventilation and blood flow might have solely influenced the formation of the epithelioid cell granulomas.

In this case, we initially suspected pulmonary carcinoid recurrence. Therefore, we did not perform preoperative diagnosis using fine‐needle biopsy because of the risk of haemorrhage. It was reasonable to perform intraoperative diagnosis because of the lower risk of uncontrolled bleeding. Because high accumulation on FDG‐PET might be confused with the presence of a malignant tumour, we believe this case has educational value.

## CONFLICT OF INTEREST

None declared.

## AUTHOR CONTRIBUTION

Shinnosuke Yokotsuka wrote the draft and Shugo Uematsu revised the manuscript. Momoka Okada, Shinnosuke Takamiya, Shinichi Ohashi, Yoko Tanaka, Kosuke Suzuki, Akihiko Kitami and Tetsuo Nemoto contributed substantially to critical review, and Tetsuo Nemoto contributed to the pathological diagnosis. All authors reviewed and approved the final version of the manuscript.

## ETHICS STATEMENT

Appropriate written informed consent was obtained for publication of this case report and accompanying images.
